# Surgical classification for large macular hole: based on different surgical techniques results: the CLOSE study group

**DOI:** 10.1186/s40942-022-00439-4

**Published:** 2023-01-30

**Authors:** Flavio A. Rezende, Bruna G. Ferreira, Emmanouil Rampakakis, David H. Steel, Michael J. Koss, Zofia A. Nawrocka, Daniela Bacherini, Eduardo B. Rodrigues, Carsten H. Meyer, Tomaso Caporossi, Tamer H. Mahmoud, Stanislao Rizzo, Mark W. Johnson, Jay S. Duker

**Affiliations:** 1 Department of Ophthalmology, Maisonneuve-Rosemont Hospital, CIUSSS de l’est d’ile de Montréal, University of Montreal, 801 Rue de la Commune est, ap 501, Montreal, QC H2V0A3 Canada; 2 Faculty of Medicine and Health Sciences, McGill University, Montreal, QC Canada; 3 Sunderland Eye Infirmary, Sunderland, and Newcastle University, Newcastle-Upon-Tyne, UK; 4 Augenzentrum Nymphenburger Höfe/Augenklinik Herzog Carl Theodor, Munich, Germany; 5 Department of Ophthalmology, Klinika Okulistyczna, Lodz, Poland; 6 Department of Neurosciences, Psychology, Drug Research and Child Health, Eye Clinic, University of Florence, Florence, Italy; 7 Department of Ophthalmology, St. Louis University, St. Louis, MO USA; 8 Augenärzte Graubünden, Davos, Switzerland; 9 Fondazione Policlínico Universitario Agostino Gemelli IRCCS, Università Cattolica del Sacro Cuore, Rome, Italy; 10 Instituto di Neuroscienze - CNR, Pisa, Italy; 11 Associated Retinal Consultants, Beaumont Neuroscience Center, Oakland University William Beaumont School of Medicine, Royal Oak, MI USA; 12 Department of Ophthalmology and Visual Sciences, University of Michigan, Ann Arbor, MI USA; 13 New England Eye Center, Tufts Medical Center, Boston, MA USA; 14 Department of Ophthalmology, Tufts Medical Center, Boston, MA USA

**Keywords:** Large macular holes, Surgical macular hole classification, Close study group

## Abstract

**Background:**

The CLOSE study group proposes an updated surgical classification for large macular holes based on a systematic review of new treatments. Recently, many new techniques have been introduced to treat large full-thickness macular holes (FTMH); although the indications are not clear. An updated surgical classification is needed to help surgical decision-making.

**Methods:**

We gathered published series by the CLOSE Study Group members and from literature search until June 2021. Techniques included: internal limiting membrane peeling (ILM peeling), ILM flaps, macular hydrodissection (macular hydro), human amniotic membrane graft (hAM), and autologous retinal transplantation (ART). Within each technique, chi-square test assessed association between the minimal linear diameter (MLD) (in µm) and closure rate; the postoperative best-corrected visual acuity (BCVA) gains were compared among groups.

**Results:**

Data extraction included 31 published articles: total of 1135 eyes. Eyes were divided into the following groups: ILM peel (n: 683), ILM Flap (n: 233), macular hydrodissection (n: 64), hAM (n: 59), and ART (n: 96). The initial BCVA and size were heterogenous between the groups. ILM peel showed the best results in large FTMH ≤ 535 µm (closure rate 96.8%); adjusted mean BCVA: 0.49 (LogMAR) with a statistical difference among groups. Large FTMH between 535 and 799 µm: ILM flap technique showed better results (closure rate 99.0%); adjusted mean BCVA: 0.67(LogMAR); also with a statistical difference. For large FTMH ≥ 800 µm more invasive techniques are required. Use of hAM, macular hydrodissection and ART showed higher closure rates for this category (100%, 83.3% and 90.5% respectively), and adjusted mean BCVA varied from 0.76 to 0.89. Although there was no statistical difference between those techniques for this group due to the smaller number of cases.

**Conclusions:**

The CLOSE study group demonstrated the potential usefulness of a new surgical classification for large FTMHs and propose OCT biomarkers for use in clinical practice and future research. This new classification demonstrated that Large (400–550 µm) and X-Large (550–800 µm) holes can be treated highly successfully with ILM peel and ILM flap techniques, respectively. Further studies are necessary for the larger FTMHs (XX-Large and Giant), using the CLOSE classification, in order to determine which technique is better suited for each hole size and characteristics.

## Introduction

Classification systems in medicine allow for a better understanding of a disease’s natural history, prognosis and outcomes, and, if accurate, assist in decision-making to improve patient quality of life.

The first classification system for macular holes (MHs) was the Gass classification [1]. Despite being developed over 30 years ago, before the advent of optical coherence tomography (OCT), the Gass classification allowed physicians to inform patients about their prognosis with observation alone. The classification’s main focus was on pathophysiology rather than surgical planning. For instance, a stage 3 MH (> 400 µm in diameter with partial posterior vitreous detachment [PVD]) could actually be larger than a stage 4 MH (> 400 µm in diameter with a complete PVD) because the only difference between these hole stages was the PVD stage.

In the early 1990s, Kelly and Wendell pioneered the use of pars plana vitrectomy (PPV) to treat MHs and reported reasonable anatomic results with removal of central vitreous, attempt at posterior hyaloid separation, and gas tamponade, and no additional maneuvers [2].

Since then, internal limiting membrane (ILM) peeling along with a myriad of other surgical techniques, including staining of the retinal surface with vital dyes (chromovitrectomy), and pharmacologic vitreolysis, has emerged [3]. Ocriplasmin (Jetrea, ThromboGenics, Iselin, NJ) is currently the only FDA-approved pharmacologic agent for MHs, but its use is limited by adverse effects. Considering this, the International Vitreomacular Traction Study Group (IVTS) introduced a new classification to identify features of MHs predictive of clinical outcomes [4]. MHs (aperture size, OCT-based measurement, parallel to the retinal pigment epithelium [RPE] plane, or of central minimal MH width) were classified as small (≤ 250 µm), medium (> 25 to ≤ 400 µm), and large (> 400 µm). The focus of the current classification scheme was to identify smaller MHs and vitreomacular adhesion sizes potentially amenable to vitreolysis with ocriplasmin [4]. The measurement used by the IVTS Group will be referred to in text as the minimal linear diameter (MLD). Neither classification systems considered either unsuccessfully treated or recurrent MHs (refractory MHs).

Currently, one of the most popular procedures for MH closure is small-gauge PPV with internal limiting membrane (ILM) peeling and gas tamponade [5]. Using this approach, Liu and co-authors confirmed the prognostic value of the IVTS classification, which achieved a nearly 100% success rate with one or more interventions for small and medium MHs, but only about an 80% closure rate and lower visual outcomes for large MHs (> 400 µm MLD) [5].

The success rate regarding hole closure is very high in small and medium holes, so it is important to determine the hole diameter at which the success rate of conventional surgery begins to decline. Two studies to date have addressed this question using sizable cohorts of patients with large MHs treated with PPV and ILM peeling [6, 7]. Both studies have found that at a MLD of about 500 µm, the closure rate declined from very high (97–98%) to about 90%. Ch’ng and colleagues reported a further reduction in the success rates to about 75% for MHs with a MLD of 630 µm [6] or greater, while Steel and co-workers found a more gradual reduction in success rates for holes with MLDs exceeding 500 µm [7]. It should be noted that because of their rarity the data reported for very large MHs with MLDs above 900 to 1000 µm is limited.

The findings of these two studies help guide clinical practice about when to use adjunctive maneuvers to maximize the chances of hole closure. Any such guidance based on MH size involves weighing the potential incremental morbidity inherent in the proposed extra procedure against the potential anatomic and visual gains. The findings suggest that for MHs with MLDs below 500 µm, the closure rate is very high, and no extra procedures are warranted; above 630 µm, it is reasonable to consider additional surgical steps to facilitate closure; and between 500 and 630 µm, adjunctive maneuvers may be indicated at the surgeon’s discretion and based on the risk–benefit ratios of the maneuvers, as well as the presence of other known risk factors for non-closure including a lower MH index (MHI), the ratio between the hole edge height and the base linear diameter (BLD) of the hole (measured by OCT), and high myopia [8, 9].

To enhance the current understanding of MH treatment outcomes, a group of experts that introduced different surgical techniques (CLOSE Study Group) convened to propose an updated classification for large MHs based on the MLD and other spectral-domain (SD)-OCT parameters. The new classification presented here is based on data that compare visual outcomes and closure rates for MHs with MLDs exceeding 400 µm that were treated with some of the newer adjunctive techniques. This classification is designed to help surgeons in the decision-making process to obtain the best anatomic and functional results for both primary and refractory (persistent or recurrent) MHs and general ophthalmologists to be aware of significant improvement in surgical outcomes of previously untreatable MHs.

The primary objective was to compare each surgical technique’s anatomic and functional results among the different MLD size groups for MHs. For each technique, the MH closure rate and improvement in the best-corrected visual acuity (BCVA) were compared according to the preoperative MH MLD. The secondary objective was to create a new surgical classification for large MHs based on the review of these studies and other SD-OCT biomarkers.

## Methods

We first gathered a group of vitreoretinal surgeons considered to be experts in the field of MH surgical treatment. The group was named the CLOSE Study Group (Classification for Large Macular Hole Studies). The group members either introduced the surgical techniques included in this study, have participated in previous macular hole classification studies or have wide surgical experience in the subject. All group interactions took place virtually and all surgeons contributed with most cases included in this analysis. Due to the recent introduction of some of the techniques, after detailed literature search, we decided to add other series that respected the inclusion/exclusion criteria listed below for the treatment of large MHs for a broader representation of our proposed classification.

### Study selection: inclusion and exclusion criteria

A literature review was conducted by authors through studies published until June 2021. The studies included should present data concerning one of the surgical techniques researched and should disclose information of the patients individually.

We included publications that contained patient-level information on the following parameters: preoperative MLD (in µm), surgical technique, closure outcomes based on the Rossi et al. classification [10], and the preoperative and postoperative logarithm of the minimum angle of resolution (logMAR) BCVA. We included refractory holes (persistent or reopened), pathologic myopia, and chronic MHs exceeding 400 µm because most large MHs are included in these groups. Although little data is available on the MHI and hole-edge configuration for MHs with MLDs over 400 µm, we added them to the proposed classification because we believe that they are relevant.

Most authors in the CLOSE Study group contributed with their own published work, sharing the data of the patients individually. We excluded studies that didn’t provide patient-level data.

Exclusion criteria included: studies that didn’t describe the technique used or didn’t provide patient-level data; studies that included other ocular pathologies such as glaucoma and retinal detachment; that could interfere with final visual acuity.

### Search methods to identify studies

Studies were searched in Pubmed, Embase, and Cochrane using the following terms: “macular holes” or “large macular holes” or “giant macular hole” and “treatment” or “pars plana vitrectomy” or “ILM peel” or “peeling” or “ILM free-flap” or “inverted ILM flap” or “macular hydrodissection” or “peri-foveal hydrodissection” or “retina expansion” or “human amniotic membrane” or “retinal graft” or “autologous retinal graft” or “autologous retinal transplantation.”

A list with the articles published through June 2021 was reviewed and duplicate titles were deleted. We also searched the reference lists of these articles and the grey literature to identify other relevant papers reporting cases for analysis.

### Data collection

Two authors (BGF and FAR) independently read the titles and abstracts of each article identified in the previous steps and excluded irrelevant reports. Finally, for the remaining studies, the same authors read the full-text articles and selected studies for inclusion based on the inclusion/exclusion criteria listed below.

Data extracted from each article included the preoperative and postoperative BCVA, MLD, surgical technique, and anatomic results on OCT. When patient-level data were unavailable in a publication, the article was excluded, or the primary authors were contacted to request their raw data. We included only full-length articles written in either English or French. Articles that used only the Gass classification [1] without providing MLD data were excluded.

### Data analysis

Within each surgical technique, the association between the groups divided by MH MLD size and closure was assessed using the chi-square test, while the postoperative BCVA was compared between the groups divided by MH MLD size using a generalized linear model adjusting for the preoperative BCVA. The MH MLD size cut-offs in µm were determined based on receiver operating characteristic (ROC) curve analysis regarding MH closure of the ILM peeling and autologous retinal transplantation (ART) surgical techniques, which accounted for 80% of all eyes included in the analysis. *P* < 0.05 was considered statistically significant. All analyses were conducted using SPSS, version 24.0 (IBM Corp., Armonk, NY).

## Results

### Study identification

**Fig. 1:**
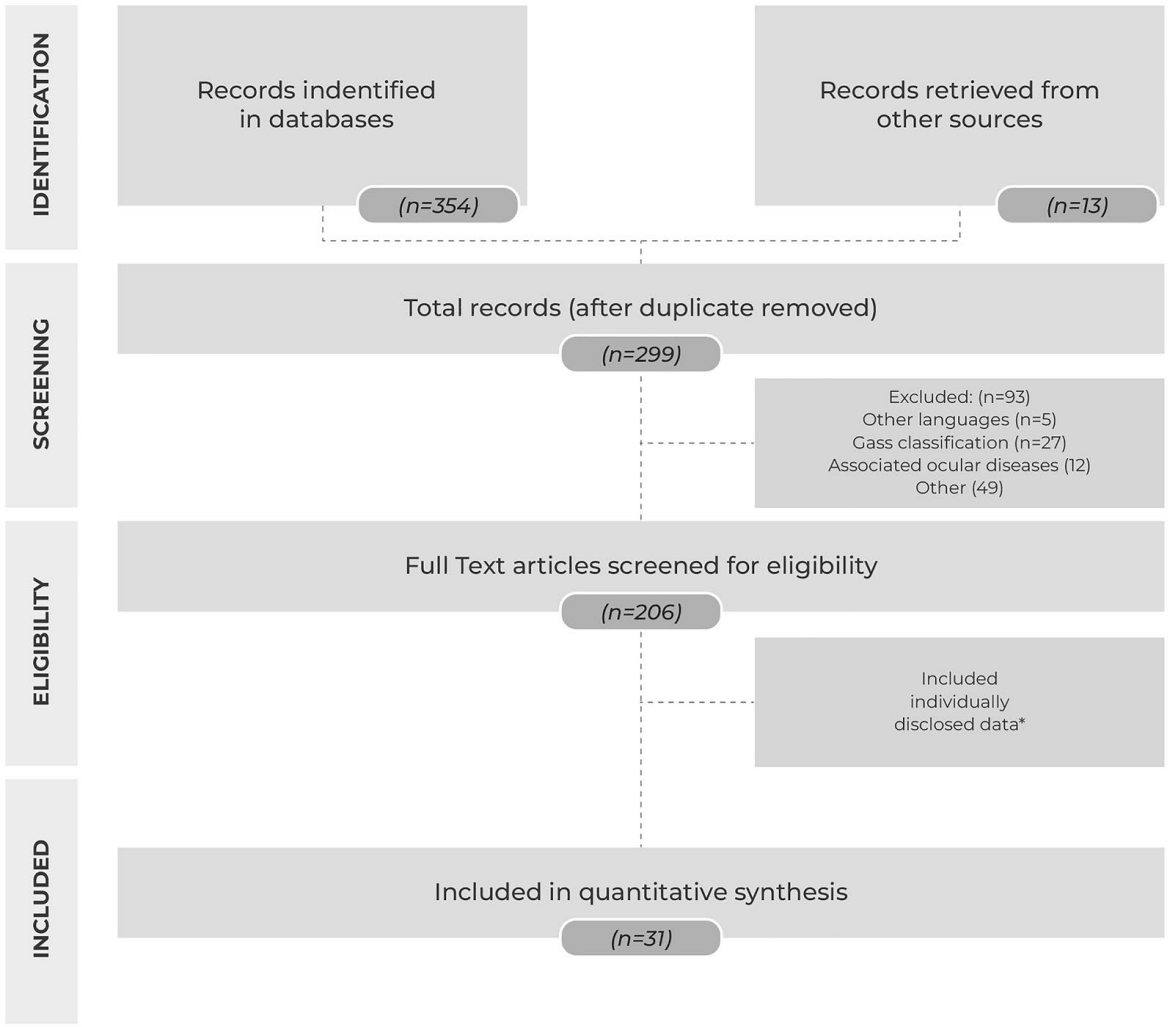
Flow diagram of the search process and study selection of articles (n = number of records in each category)

In the initial database search, we identified 354 records by searching Pubmed, Embase, and Cochrane. All articles were reviewed, and 13 other records were added via the reference search. After excluding duplicate reports, 299 records remained (Fig. 1).

The title and abstract of each article were reviewed and the following were excluded: texts written in languages other than English and French; texts that classified the MHs exclusively based on the Gass criteria and did not include the MLD; studies that included other ocular pathologies such as glaucoma, retinal detachment, macular dystrophies and trauma (high myopia was not excluded); and articles unrelated to our analyses.

In total, 206 manuscripts were read fully. Papers that disclosed data for each individual patient and those with the MHI and hole-edge configuration data were included in the quantitative analysis or to help build the proposed classification. Authors of manuscripts that did not include this information but were deemed relevant were contacted and provided their raw data to make this study possible.

Thirty-one studies ultimately met all inclusion/exclusion criteria (Fig. 1). Members of the CLOSE study group participated in thirteen of these studies, representing 1044 eyes (82.5%) from the 1265 eyes included in the analysis. [7, 12–16, 23–25, 31, 39–41].

### Data extraction

For each retained study, first author, publication year, number of patients, age and gender of patients (when available), MLDs exceeding 400 µm (measured using SD-OCT, except in one earlier publication that used time-domain OCT), preoperative BCVA, surgical technique, anatomic outcomes (any full-thickness foveal discontinuity was considered a persistently open hole), and postoperative BCVA (the final VA provided by the authors) were compiled.

The VA was converted to logMAR using the conversion system proposed by Holladay [11]. The VA was measured preoperatively and postoperatively. We included the final BCVA measured in each of the studies up to 3 years postoperatively (ranging from 4 weeks [7] to 3 years. [12]).

Case series and case reports that provided complete data for each MH case were included. For articles that did not include the needed data, first authors were contacted and asked to provide their raw data. Most coauthors included in the CLOSE Study Group contributed with their own personal published data.

The cases were classified according to the surgical technique as follows: autologous retinal transplantation (ART); macular hydrodissection or macular expansion technique (macular hydro); ILM peeling (ILM peel); ILM flaps, including inverted flap techniques and free ILM flap techniques (ILM flap) and human amniotic membrane graft (hAM).

The 31 studies included in the statistical analysis of the surgical treatment of MHs with a MLD exceeding 400 µm are summarized in Table 1.

**Table 1:** Studies Included in the Analysis

First author, year	Study type	no. eyes	Mean age (years)	MLD (µm)	Pre-VA (logMAR)	Post-VA (logMAR)	Surgical technique	Closure
Moysidis et al. [12]	Multicenter, retrospective, global consortium	130	63 (± 6.3)	840	1.37 (± 0.12)	1.05 (± 0.09)	ART	115/130 (88.4%)
Meyer et al. [13]	Multicenter, retrospective, case series	41	NA	1,276	1.22 (± 0.45)	0.72 (± 0.31)	Macular hydro	35/41 (85.4%)
Michalewska et al. [14, 15]	Prospective, randomized clinical trial [46] Prospective comparative interventional [47]	157	67.1 (± 10.2)	594 (± 147.0)	0.99 (± 0.35)	0.65 (± 0.40)	ILM flap	154/157 (98.1%)
Giansanti et al. [16]	Retrospective, consecutive, nonrandomized	8	74 (± 4.8)	436 (± 46.9)	0.81 (± 0.16)	0.66 (± 0.09)	ILM flap	8/8 (100%)
Steel et al. [7]	Multicenter, retrospective	636	69.5 (± 7.5)	525 (± 104)	1.04 (± 0.42)	0.55 (± 0.34)	ILM peel	47/637 (92.6%)
Kumar and Yadav [17]	Retrospective, case series	25	56.8 (± 14.9)	501 (± 162)	1.04 (± 0.29)	0.60 (± 0.29)	ILM peel	25/25 (100%)
Kikushima et al. [18]	Single-center, prospective interventional case series	20	63.5 (± 12.6)	373 (± 139)	0.515 (± 0.28)	NA	ILM peel	20/20 (100%)
Frisina et al. [19]	Prospective, interventional case series	10	NA	230 (± 117)	1.06 (± 0.08)	0.56 (± 0.22)	Macular hydro	9/10 (90%)
Primavera et al. [20]	Single-center, case series	5	67.4 (± 5.9)	666 (± 167)	1.08 (± 0.37)	0.52 (± 0.15)	ILM flap	5/5 (100%)
Wong et al. [21]	Retrospective, interventional case series	16	72.3 (± 8.9)	739 (± 62)	1.36 (± 0.45)	0.9 (± 0.24)	Macular hydro	14/16 (87.5%)
Liu et al. [22]	Case report	1	68	NA	1.4	0.4	ART	1/1 (100%)
Rizzo et al. [23]	Prospective, interventional case series	8	69.5 (± 14.1)	578 (± 170.6)	1.49 (± 0.50)	0.64 (± 0.25)	hAM	8/8 (100%)
Grewal and Mahmoud [24]	Case report	1	50	1100	1	0.6	ART	1/1 (100%)
Caporossi et al. [25]	Prospective, consecutive, interventional	16	68.3 (± 11.4)	716 (± 355)	0.94 (± 0.24)	0.67 (± 0.27)	hAM	15/16 (93.75%)
Fung et al. [26]	Retrospective, interventional, single center	8	67.5 (± 6.6)	821 (± 361)	1.04 (± 0.19)	0.69 (± 0.21)	ILM flap	7/8 (87.5%)
Chang et al. [27]	Retrospective, case series, single center	10	64.9 (± 11.5)	1404 (± 562.9)	1.72 (± 0.58)	0.89 (± 0.55)	ART	9/10 (90%)
Chen [28]	Retrospective case series	17	63 (± 11.6)	500	1.22 (± 0.59)	0.64 (± 0.4)	ILM flap	16/16 (100%)
Dai et al. [29]	Prospective, interventional case series	13	55.3 (± 19.6)	814 (± 255)	1.15 (± 0.21)	0.99 (± 0.17)	ILM flap	13/13 (100%)
de Novelli et al. [30]	Prospective, interventional case series	10	60.8 (± 14.3)	669 (± 170.0)	1.30 (± 0.47)	0.99 (± 0.33)	ILM flap	10/10 (100%)
Tanaka et al. [31]	Retrospective, case series single center	7	71.4 (± 8.6)	661 (± 103.7)	1.23 (± 0.21)	0.84 (± 0.49)	ART	7/7 (100%)
Kang et al.^.^ [32]	Retrospective case series	29	63.5 (± 8.6)	537 (± 214.1)	0.92 (± 0.33)	0.55 (± 0.28)	ILM peel	19/29 (65.5%)
Chen et al. [33]	Prospective, interventional, single center	8	65.(± 9.0)	628 (± 172.4)	1.28 (± 0.39)	0.63 (± 0.22)	ILM flap	8/8 (100%)
Kusuhara et al. [34]	Prospective interventional case series	25	64.9 (± 6.3)	315 (± 141.1)	0.90 (± 0.31)	0.37 (± 0.37)	ILM peel	25/25 (100%)
Mahalingam et al. [35]	Prospective interventional case series	5	67 (± 5.4)	811 (± 106.2)	1.26 (± 0.30)	1.10 (± 0.23)	ILM flap	5/5 (100%)
Shakya et al. [36]	Case series	10	NA	1039 (± 291.2)	1.29 (± 0.25)	0.93 (± 0.14)	ILM flap	10/10 (100%)
Wong [37]	Case series	3	81 (± 9.0)	717 (± 23.9)	1.60 (± 0.69)	0.87 (± 0.12)	Macular hydro	3/3 (100%)
Wu et al. [38]	Retrospective, consecutive, interventional case series	6	59 (± 9.9)	538 (± 202.6)	1.47 (± 0.31)	1.08 (± 0.53)	ART	4/6 (66.7%)
Ferreira et al. [39]	Retrospective chart review	19	66 (± 15.0)	856 (± 459.3)	1.1 (± 0.44)	1.1 (± 0.72)	hAM	19/19 (100%)
Caporossi et al. [40]	Prospective, interventional, comparative	20	68 (± 12.3)	789 (± 155.7)	1.1 (± 0.48)	0.54 (± 0.14)	hAM	20/20 (100%)
Meyer et al. [41]	Case report	1	83	1444	3	1	Macular hydro	1/1 (100%)
Total		1265						

*NA* not applicable, *ILM* internal limiting membrane, *macular hydro* macular hydrodissection, *VA* preoperative visual acuity (logMAR), *post-VA* postoperative visual acuity (logMAR), *logMAR* logarithm of the minimum angle of resolution, *hAM* human amniotic membrane, *ART* autologous retinal transplantation

We collected data from 1265 eyes. Some eyes were subsequently excluded because of missing information concerning the preoperative MLD. After exclusion, a total of 1135 eyes were categorized (Table 2) based on the surgical technique and MH MLD (µm).

**Table 2:** Macular hole groups included in the statistical analysis

Technique size (µm)	ILM peel	ILM flap	Macular hydro	hAM	ART	Total
> 400 to 535	406	77	1	12	5	501
535 to 799	265	117	27	24	49	482
800 to 1000	10	29	5	16	19	79
> 1000	2	10	31	7	23	73
Total	683	233	64	59	96	1135

*ILM* internal limiting membrane, *hAM* human amniotic membrane, *ART* autologous retinal transplantation, *macular hydro* macular hydrodissection

### MH groups classification

**Fig. 2:**
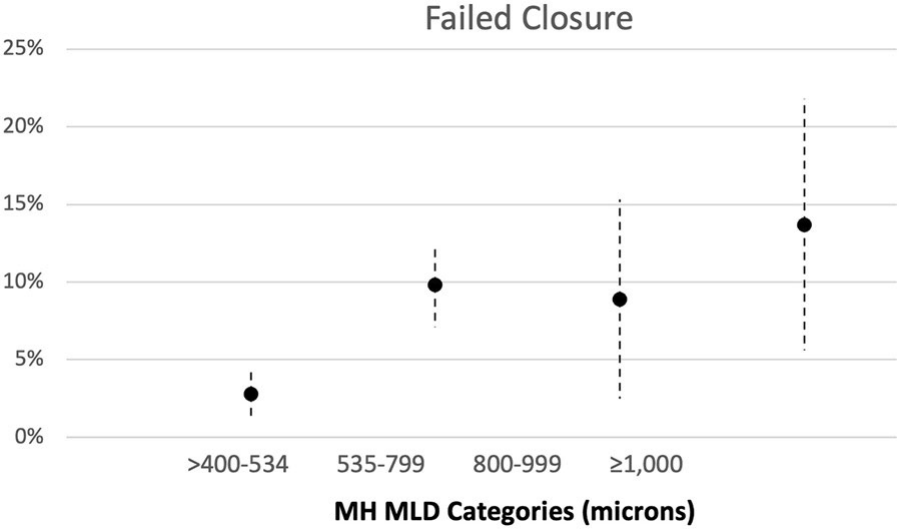
Correlation of macular hole (MH) categories (divided into 4 groups) based on the preoperative minimum linear diameter (MLD) and percentage of postoperative failure to close

**Fig. 3:**
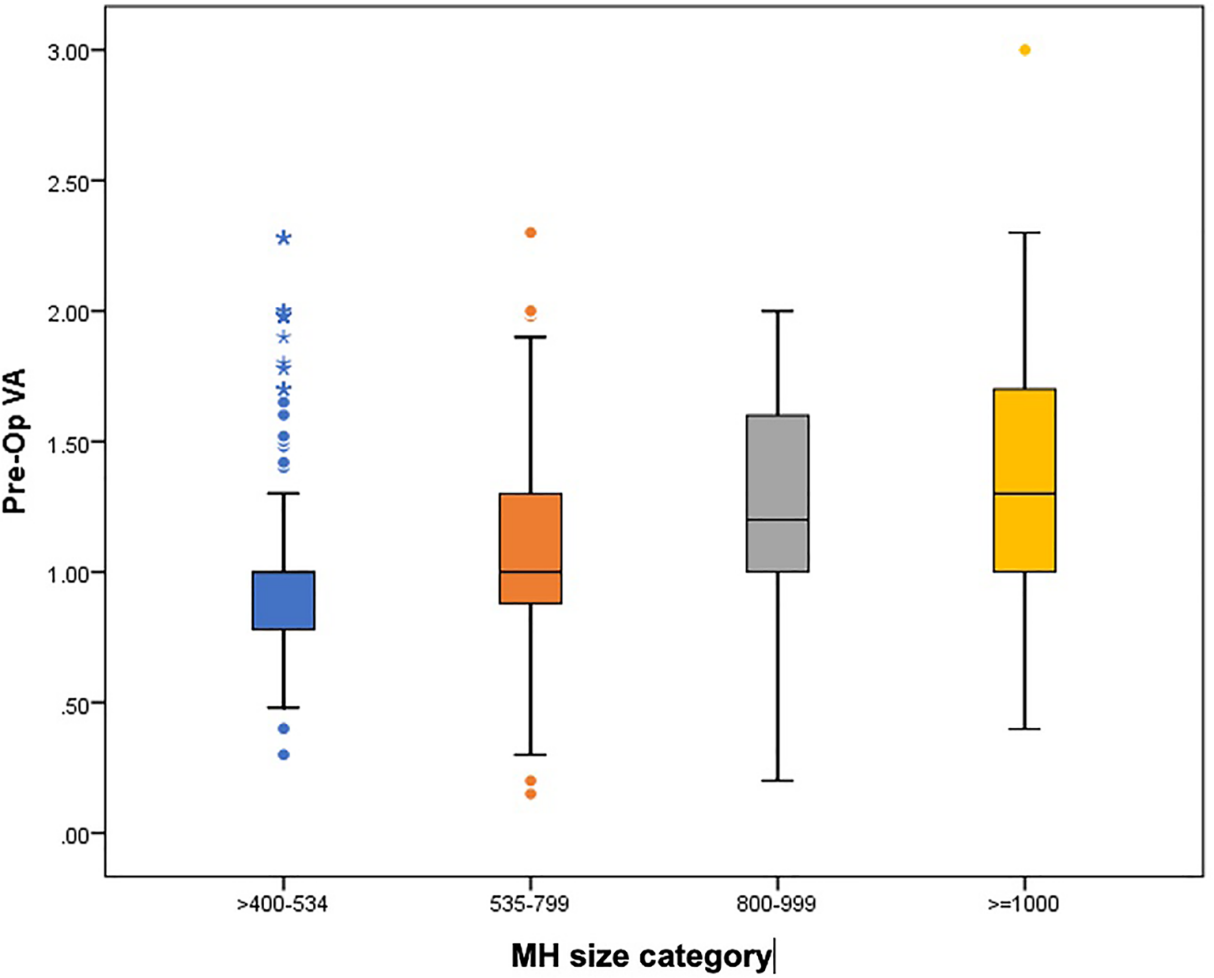
Association between the preoperative macular hole (MH) minimum linear diameter (MLD) size measured by optical coherence tomography (in µm) and the preoperative logarithm of the minimum angle of resolution visual acuity (pre-op VA)

The authors classified the MH groups according to the preoperative MLD. The MH size cut-offs were: over 400–535 µm, 536–799 µm, 800–999 µm, and 1,000 µm or larger (Fig. 2). The cut-offs of 535 µm and 800 µm were determined based on the ROC curve analysis of MH closure achieved by the ILM peel and ART surgical techniques, respectively. The cut-off of holes exceeding 400 µm was used based on the IVTS results [4] and the cut-off of 1000 µm was added based on expert clinical opinion to account for giant MHs. Based on this distribution, the larger the MLD size group, the worse the preoperative BCVA (*P* < 0.001) (Fig. 3).

### Success rates by MLD group in each surgical technique

**Fig. 4:**
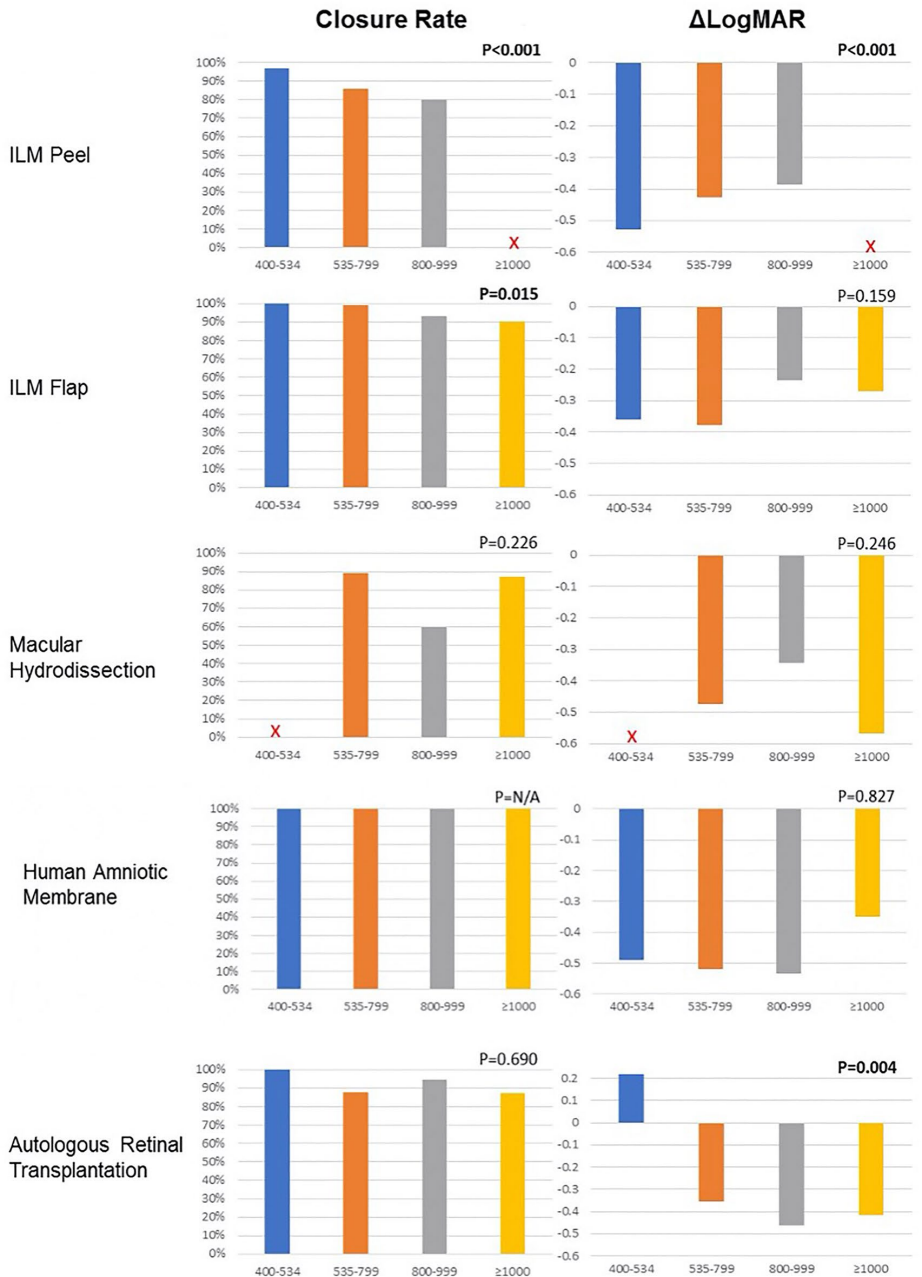
The graphs show the outcomes for each technique. Outcomes are categorized by the preoperative minimum linear diameter (MLD) measured on optical coherence tomography. The graphs on the left show the percentages of macular hole (MH) closure for each MLD size group. The graphs on the right show the improvement in the logarithm of the minimum angle of resolution (logMAR) (ΔlogMAR) visual acuity for MHs in each MLD size group. The X indicates MLD size groups with an insufficient number of eyes to be included in the analysis. ILM = internal limiting membrane

The outcomes (MH closure rate and postoperative BCVA change, expressed as ΔlogMAR) for each surgical technique were analyzed based on the preoperative MLD size group (Fig. 4).

#### ILM peeling

ILM peeling showed the best results for MHs with a MLD ranging from 400 to 535 µm, with a closure rate of 96.8%; the closure rate decreased significantly for the remaining groups down to 80% for the 800- to 999 µm group (*P* < 0.001). Insufficient data were available for MHs of 1,000 µm and larger. The average VA gain in the MHs ranging from > 400 to 535 µm was around five lines, but, consistent with lower closure rates, decreased significantly with increasing MLD size (*P* < 0.001) (Fig. 3).

#### ILM flap

Free ILMs and inverted flap techniques were pooled in this surgical category using either approaches that covered or filled the hole to achieve a significant number of cases. Subcategories within this technique were not possible, because each study described a slightly different procedure. ILM flap techniques had higher closure rates and better VA gains for the first two groups (> 400 to 535 µm and 536 to 799 µm) compared with the two larger groups (*P* = 0.015) (Fig. 3).

#### Macular hydrodissection

This technique included procedures involving subretinal injection of saline solution in the peri-hole region to detach the edges of large MHs from the RPE. Outcomes with this technique trended better for holes with MLDs of 535 to 799 µm and 1000 µm and larger, with closure rates of 88.9% and 87.1%, respectively, but did not differ significantly from outcomes in holes ranging from 800 to 999 µm (*P* = 0.226). There was insufficient data for the MHs ranging from over 400 to 535 µm. A mean BCVA gain of about five lines was seen in the MHs 1000 µm and larger (*P* = 0.246).

#### Human amniotic membrane (hAM) graft

The hAM procedure achieved a MH closure rate of 100% regardless of MLD size. The mean BCVA gain was approximately five lines for holes between 800 and 999 µm or smaller and 3.5 lines for holes 1000 µm and larger (*P* = 0.827) (Fig. 4).

#### Autologous retinal transplantation

ART showed consistent anatomic success for all MLD sizes and achieved an 87% full-thickness MH (FTMH) closure rate in the 1000 µm and larger group. The VA did not improve for holes greater than 400 to 535 µm and showed a mean BCVA gain of about four lines for holes 1000 µm and larger (*P* = 0.004) (Fig. 4).

### VA Outcomes

The overall functional outcomes of the different techniques, irrespective of MLD size, are expressed as the mean difference between the preoperative and postoperative BCVAs (ΔlogMAR) in Table 3.

**Table 3:** Correlation of preoperative MLD (measured in µm) with visual acuity gain in logMAR (ΔlogMAR) for each surgical technique

Technique Δ MLD (µm)	hAM	Macular Hydro	ILM Flap	ART	ILM Peel
> 400 to 534	− 0.4902	− 0.2712	− 0.3602	0.2202	− 0.5293
535 to 799	− 0.5177	− 0.4748	− 0.3778	− 0.3561	− 0.4248
800 to 999	− 0.5342	− 0.3441	− 0.2338	− 0.4633	− 0.3858
≥ 1000	− 0.3497	− 0.5664	− 0.2694	− 0.4178	− 0.0309
P Value	P = 0.827	P = 0.246	P = 0.159	P = 0.004	P < 0.001

*MLD* minimal linear diameter, *hAM* human amniotic membrane graft, *ILM* internal limiting membrane, *ART* autologous retinal transplantation, *logMAR* logarithm of the minimum angle of resolution, *macular hydro* macular hydrodissection

## Discussion

Mahmoud and Thompson highlighted the need for a new classification of larger MHs because of the recent introduction of effective new treatments for this condition [42]. A recent retrospective series [43] on refractory MHs illustrated the need for a new classification. The authors could not establish any difference in outcomes between a revisional surgery with tamponade alone versus adjuvant manipulation (free flap, macular hydrodissection, hAM, ART, or autologous blood). They advised that their results be interpreted with caution due to the significant differences in MLDs between their groups (415 ± 205 µm vs. 546 ± 188 µm) [12]. The latest meta-analysis of studies investigating refractory MHs [13] reported clinically meaningful VA improvement in over half of eyes with reoperated MHs, although some studies included in their analysis used Gass staging alone and failed to disclose the preoperative MLDs [1]. Reviews on the strengths and challenges of newer surgical techniques did not analyze surgical outcomes by MH size exceeding 400 µm [44–47].

The first attempt at establishing a new classification scheme for large MHs was the Manchester Large Macular Hole Study [6]. For the first time, the authors addressed the differences in surgical outcomes among different hole sizes with MLDs over 400 µm and proposed a surgical classification based on their results. However, they included only eyes undergoing ILM peeling and grouped the holes into only two size categories: over 250 to 650 µm (medium MHs) and over 650 µm (large MHs).

**Fig. 5:**
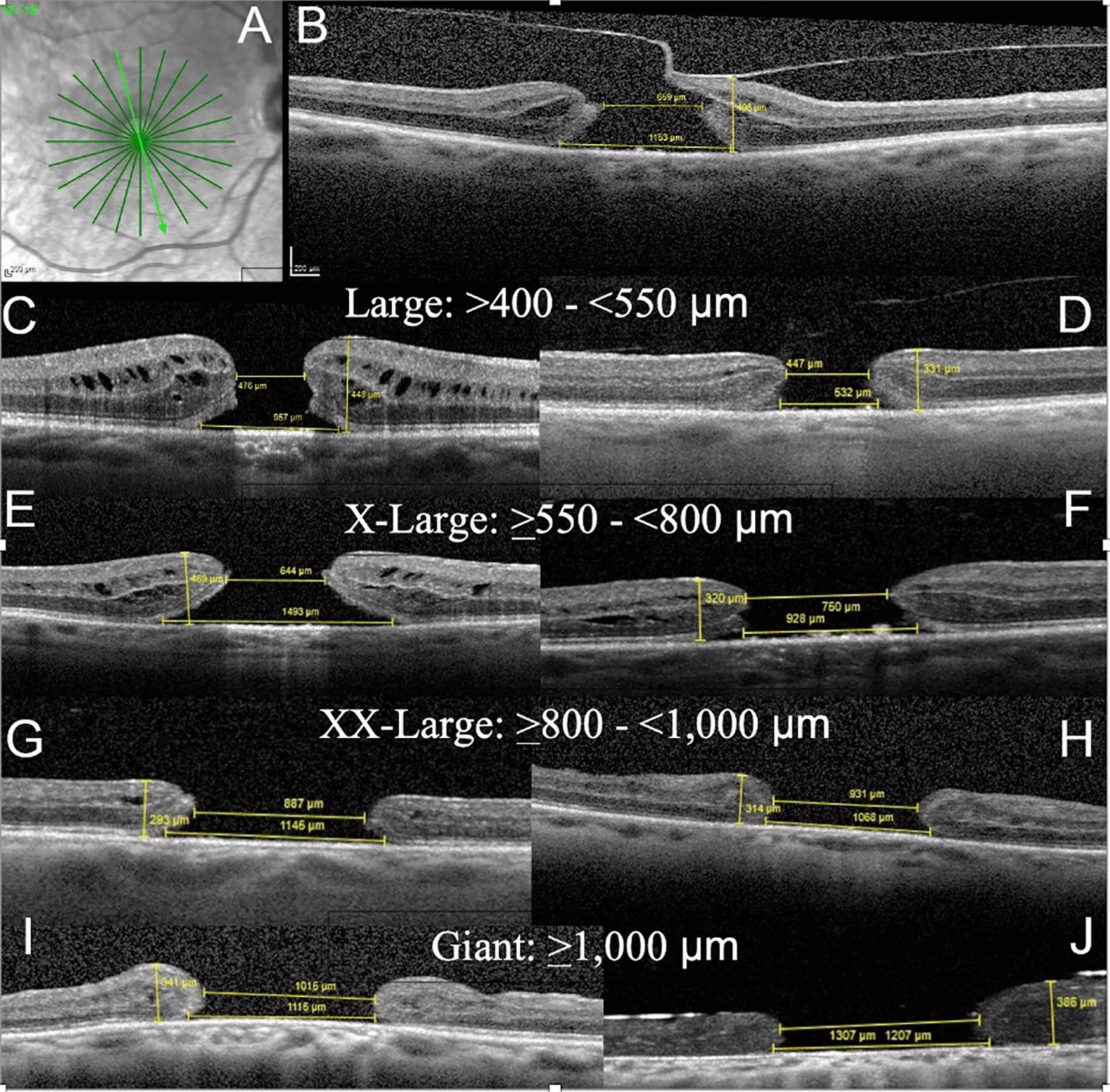
Spectral-domain optical coherence tomography (OCT) radial scans of full-thickness macular hole (FTMH) over 400 µm in minimum linear diameter (MLD) show each hole size group. The measurements of the MLD, base linear diameter (BLD), and hole height (at the highest point around the hole) are shown. The macular hole index (MHI) is calculated by the ratio of height to BLD. The edge configuration is described as having a fluid cuff or being flat and the presence/absence of edematous cysts. Vitreomacular traction (VMT) and epiretinal membranes (ERMs) also are described. With increasing hole size, the fluid cuff and edematous cysts tend to disappear. **A** Radial scans centered on the FTMH are used to detect the largest MLD and presence of VMT. **B** Only one of the radial scans from A shows focal VMT in this primary X-large (XL) hole with a MLD of 659 µm, a BLD of 1153 µm, height of 496 µm, MHI of 0.43 with a fluid cuff and edematous cysts, and no ERM. **C** A primary large FTMH with a MLD of 476 µm, BLD of 957 µm, height of 448, MHI of 0.47, with a cuff and edematous cysts, no VMT, and no ERM. **D** A primary large FTMH with a MLD of 447 µm, BLD of 532 µm, height of 331 µm, MHI of 0.62, no cuff, no cysts, no VMT, and no ERM. **E** A primary XL hole with a MLD of 644 µm, BLD of 1493, height of 469 µm, MHI of 0.31, with cuff and cysts, no VMT, and no ERM. **F** A refractory XL hole with a MLD of 750 µm, BLD of 928 µm, height of 320 µm, MHI of 0.35, no cuff, no cysts, no VMT, and no ERM. **G** A refractory XXL hole with a MLD of 887 µm, BLD of 1145 µm, height of 293 µm, MHI of 0.26, with a cuff, no cysts, no VMT, and no ERM. **H** A refractory XXL hole with a MLD of 931 µm, BLD of 1068 µm, height of 314, MHI of 0.29, no cuff, no cysts, no VMT, and no ERM. **I** A refractory giant hole with a MLD of 1015 µm, BLD of 1115 µm, height of 341 µm, MHI of 0.31, no cuff, no cysts, and no VMT or ERM. **J** A refractory giant hole under silicone oil tamponade with a MLD of 1207 µm, BLD of 1307 µm, height of 386 µm, and no cuff, cysts, VMT, or ERM

Here, the CLOSE Study Group proposes a surgical classification for MHs based on the MLD data identified by the current systematic review results and the inclusion of other potentially important information on hole-edge configuration, MHI, vitreomacular traction (VMT), and epiretinal membranes (ERM) (Table 4, Fig. 5). In addition to MLD data, the MHI was included because it has also been shown to be a predictor factor for MH closure, although not used consistently in many studies [8, 34]. Although not addressed in this report, we keep the small and medium MHs criteria and the presence/absence of VMT/ERM as proposed by the IVTS classification, because its prognostic value was established. For MLDs smaller than 400 µm, very high success rates can be achieved with vitrectomy with or without ILM peeling [4, 5].

**Table 4:** Proposed CLOSE Study Group Surgical Classification for Macular Holes Based on the Minimum Linear Diameter (µm), Hole-Edge Configuration, Presence/Absence of Edematous Hole Edges, Macular Hole Index, Presence of Vitreomacular Traction, and Epiretinal Membrane

Group	Hole Size (MLD) (µm)	Hole-edge configuration	edematous edges	MHI (Height/BLD)	VMT	ERM
VMT					Focal/ Diffuse	
Small	< 250	Cuff or flat	Yes/no		Yes/no	Yes/no
Medium	> 250 to ≤ 400	Cuff or flat	Yes/no		Yes/no	Yes/no
Large	> 400 to ≤ 550	Cuff or flat	Yes/no		Yes/no	Yes/no
X-Large	> 550 to ≤ 800	Cuff or flat	Yes/no		Yes/no	Yes/no
XX-Large	> 800 to ≤ 1000	Cuff or flat	Yes/no		Yes/no	Yes/no
Giant	> 1000	Cuff or flat	Yes/no		Yes/no	Yes/no

*MLD* minimum linear diameter, *MHI* macular hole index, *BLD* base linear diameter, cuff = separation of photoreceptors at hole edges from the retinal pigment epithelium (RPE) with difference between MLD and BLD ≥ 200 µm; flat no separation of photoreceptors from RPE or minimal separation with difference between MLD and BLD < 200 µm; edematous edges  presence of multiple cystoid cavities at hole edges; *VMT* vitreomacular traction; focal ≤ 1500 µm; diffuse   > 1500 µm; *ERM* epiretinal membrane

The current pooled analysis of surgical outcomes for large MHs (MLD > 400 µm) showed for the first time the potential benefits of dividing large MHs into subgroups by size. Clinically, it is important to perform dense radial scans to properly determine the hole dimensions and identify VMT (Fig. 5) [48]. For ease of remembering the different groups in everyday use, we recommended that the CLOSE Study Group classification define the large MH group as over 400 to 550 µm (instead of 535 µm as in our systematic review). Two previous large series that included only ILM peeling cases also showed the 550 µm MLD to be close to a common cut-off point [7, 49].

Our systematic review showed that the preoperative BCVA, a known predictive factor for good functional outcomes [50, 51], decreased significantly with each group with enlarging MLD hole size (Fig. 3) and that the closure rate for wide ILM peeling also is inversely proportional to MH size, ranging from around 97% for the large group (> 400 to 550 µm) down to 80% for the XX-large (XXL) group (> 800 to 999 µm). Another important finding was that visual gains (logMAR) with ART occurred in the XXL and giant (≥ 1000 µm) groups but not the large group (although few cases were included in that group). The hAM and ILM flap techniques had closure rates that were least affected by the MLD; the BCVA gains with hAM seemed to be affected less by hole size than with the ILM flap procedure. Importantly, BCVA gains after macular hydrodissection were marked for the groups ranging from XL (> 550 to 800 µm) to giant despite a lower closure rate for XXL holes (Fig. 4).

Figure 5 shows examples of MH sizes in each MLD group and the differences in hole-edge configurations, BLD, and MHI. As MHs enlarge, especially in refractory cases, the hole edges flatten, and edematous cystoid spaces are scarce. The change in MH pattern with increasing size may eventually prove relevant to the choice of technique/tamponade. Figure 5 also shows the importance of not categorizing all large MHs in one group as in the previous Gass and IVTS classifications [1, 4]. To correlate our proposed classification with these two previous schemes, we show them side by side in Table 5.

**Table 5:** Correlation between gass, international vitreomacular traction study, and the updated classification systems for large macular holes

FTHM stages based on gass classification [1] 1995	International vitreomacular traction study classification system [3]	CLOSE study group updated classification for FTMH based on minimum linear diameter (µm)	
Stage 0	VMA	VMA	
Stage 1: impending macular hole	VMT	VMT	
Stage 2: small hole	Small (≤ 250 μm) or medium (> 250 to ≤ 400 μm) FTMH with VMT	Small	≤ 250 µm
Stage 3: large hole	Medium or large (> 400 μm) FTMH with VMT	Medium	> 250 to ≤ 400 µm
Stage 4: FTMH with PVD	Small, medium, or large FTMH without VMT	Large	> 400 to ≤ 550 µm
		X-large	> 550 to ≤ 800 µm
		XX-large	> 800 to ≤ 1000 µm
		Giant	> 1000 µm

*VMA* vitreomacular adhesion, *VMT* vitreomacular traction, *FTMH* full-thickness macular hole, *PVD* posterior vitreous detachment

An important objective of the CLOSE Study was to introduce a new classification system for primary or refractory (persistent or reopened) MHs with MLDs exceeding 400 µm based on outcomes of various surgical techniques. We believe it will be helpful for future studies to use this classification in their analyses and for general ophthalmologists and retina specialists to use in daily practice for better referral and management, respectively, of previously considered untreatable MHs. A secondary objective was to propose other simple parameters (BLD, MHI, and hole-edge configuration) that should be documented in studies of MHs with the goals of increasing understanding and hopefully improving surgical outcomes. The design of the current systematic review is unsuitable for comparing outcomes between the surgical techniques but is useful for assessing the results of each technique for different hole sizes (Fig. 3).

In this review, we used MLD (spectral/swept-source OCT), the same parameter used by the IVTS classification [4], to stratify the MHs because it is simple to measure, has been used in most published series, and has been consistently correlated with the closure rate and visual improvement in primary and refractory holes of different sizes [52–54]. Although many of the reviewed studies did not include additional data, we believe that information such as the BLD, hole-edge configuration (elevated with a subfoveal cuff, perifoveal cysts, or flat) [55], indices such as the MHI [8], area or volumetric data [56, 57], presence of an ERM [58], axial length [9], measurements of the ellipsoidal zone (EZ) and external limiting membrane (ELM) continuity [59], a hypertransmission signal on SD-OCT [60], enface OCT [61], OCT-angiography, microperimetry [62], adaptive optics [40], and subfoveal RPE health and association with other conditions (e.g., pathologic myopia, ischemic retinopathies, and degenerative/inherited conditions) may contribute to our understanding of hole closure and visual recovery.

Current concepts of MH closure configurations have evolved recently due to interest in new surgical techniques and improved OCT technologies. Imai and colleagues (time-domain OCT) [63] and Michalewska and co-workers (SD-OCT) [64] described different OCT patterns of hole closure: the U-type (fully closed), V-type (thin glial plug closing the hole), and W-type (open hole with flat edges). Kang and co-authors (time-domain OCT) simplified closed holes into type 1, complete closure, and type 2, flat edges with an open hole [34]. These classifications focused primarily on the inner foveal contour and included only eyes undergoing ILM peeling. An updated hole closure pattern classification based on SD-OCT findings has been proposed recently by Rossi et al. [10] as follows: type 0, an open hole with bare RPE characterized by: (A) flat edges, (B) elevated edges with perifoveal cuff, and (C) hydrated/edematous edges; type 1, a closed hole characterized by: (A) integrity of both the inner and outer fovea, (B) discontinuous outer fovea, and C) discontinuous inner fovea; and type 2, a hole closed by tissue placed into the hole characterized by: (A) a tissue plug filling the entire hole, (B) integrity of the inner fovea with a tissue plug in the outer fovea, (C) integrity of the outer fovea with a tissue plug in the inner fovea, (D) and a bridging tissue plug with a discontinuous inner and outer fovea. Unsurprisingly, the authors reported better visual gains in types 1A, 1C, and 2C in which the EZ and ELM were partially/totally restored, observed as continuous on SD-OCT [10].

Park and colleagues [59] elegantly demonstrated the differences between ILM flaps that cover the MH and those that fill the hole. Their small case series showed that despite a 100% anatomic success rate with each technique, eyes in the ILM cover group had significantly better visual gains due to higher rates of EZ/ELM continuity, which was not obtained in any eyes in the ILM filled group [59]. Baumann and co-workers compared ILM peeling with ILM flap covering for MHs exceeding 400 µm, further dividing the EZ/ELM layer integrity into grade 0, absent; grade 1, partially restored but disrupted; and grade 2, fully restored and continuous [65]. Rossi and co-authors [10] confirmed this, which may explain why the current review found that despite very high success rates for all hole sizes with the ILM flap techniques, the visual gains were lower. Most published reports did not discriminate one technique from the other and therefore grouped ILM covering and ILM filling techniques together. These observations, combined with a growing understanding about glial plug formation and different types of Müller cells involved in hole formation and healing (regular/irregular foveal regeneration) [58, 66–69], should facilitate the choice of surgical techniques that will close large MHs with a smaller glial plug, increase EZ/ELM continuity, and better visual outcomes.

Important data missing in most published series were those describing the hole-edge configuration for primary or refractory MHs over 400 µm [15]. Although MLD, BLD, and MHI (ratio of hole height to BLD) appear to be the best parameters for predicting hole closure and visual gains, they do not adequately describe the hole-edge configurations [10, 63, 64]. The hole edges can be symmetric or asymmetric (have the same or different configuration on each side) and should be described as flat, have a fluid cuff (elevated edges, separation of foveal photoreceptors from the RPE), and/or edematous (cystoid spaces). Although it is often the case, edema is not always associated with a fluid cuff, that is, a hole can be edematous and flat meaning without fluid cuff around the hole (Fig. 5). Hillenkamp et al. [55] and Baumann et al. [70] have shown the importance of hole-edge configuration and MHI in revision surgeries for refractory holes using autologous blood products and fluid-gas exchange with/without ILM peeling extension, respectively. Flat edges and lower MHIs had significantly poorer outcomes compared to edematous edges with a fluid cuff. We recommend that future studies include information on hole-edge configuration for each patient to increase the understanding of its impact on the treatment outcomes of different hole sizes.

The main limitation of the current study was the low incidence of XXL (> 800 to 999 µm) and giant (≥ 1000 µm) MHs. Further, most currently performed techniques included in this analysis are fairly new, so relatively few published series are available and many have short follow-up [7]. As noted previously, most ILM flap studies did not differentiate covering versus filling techniques; also some studies used adjuncts techniques as autologous blood plug, together with other techniques [22, 38]. In most instances, macular hydrodissection, hAM, and ART were used for refractory MHs while ILM peeling and ILM flaps were applied for primary MHs. The hAM studies also varied between placing the membrane inside versus over the hole and included different types and sizes of amniotic membrane that can affect the choice of placement location and tamponade. Most ART series included grafts placed inside the hole and grafts placed partly inside and partly outside the hole, different graft donor sites, and different types of tamponade. It remains unclear whether the specific techniques used in these two latter approaches affect the surgical reproducibility and outcomes. Our classification also may not apply to all types of refractory MHs, such as those complicating myopic degeneration associated with posterior staphyloma and longer axial lengths (> 26 mm) that may interfere with reliability of OCT measurements [71], or those associated with retinal detachment, for which other techniques like macular buckling [72] or scleral imbrication [73] may be indicated. These conditions are addressed in the classification proposed by Parolini and co-authors [74].

Another limitation of this study is that we couldn’t include all described alternative techniques, such as autologous lens capsular flap, arcuate retinotomy, perifoveal radial incisions and use of heavy silicone oil, due to the lack of sufficient published data. The use of the CLOSE classification is not to be limited by the techniques included in this study, but rather to be applied by surgeons using any surgical technique.

We believe that this new surgical classification of large MHs will facilitate a well-designed prospective trial comparing outcomes of different surgical techniques for each hole size. The classification also may contribute to the development of a future more reliable artificial intelligence data source. General ophthalmologists and retina specialists can be better informed about the surgical prognosis of each hole size and characteristics. Finally, since some surgical procedures used to repair large and refractory holes remain technically challenging, future research efforts to determine which surgical technique is most beneficial for a specific hole size and edge configuration may help avoid surgery that is both technically difficult and less effective for individual patients.

In conclusion, we demonstrated the potential of a new surgical classification for MHs exceeding 400 µm in diameter and proposed documentation of other SD-OCT biomarkers for use in clinical practice and future research. Our systematic review also provides evidence that most MHs over 400 µm in diameter can be closed anatomically with significant visual gains, regardless of their size, chronicity, or previous surgical failures.

## Data Availability

The datasets used and analysed during the current study are available from the corresponding author on reasonable request.

## Abbreviations

ILM peeling: Internal limiting membrane peeling
Macular Hydro: Macular hydrodissection
hAM: Human amniotic membrane graft
ART: Autologous retinal transplantation
MLD: Minimal linear diameter
BCVA: Best-corrected visual acuity
